# Long-term consequences of adolescent cannabinoid exposure in adult psychopathology

**DOI:** 10.3389/fnins.2014.00361

**Published:** 2014-11-10

**Authors:** Justine Renard, Marie-Odile Krebs, Gwenaëlle Le Pen, Thérèse M. Jay

**Affiliations:** ^1^Laboratoire de Physiopathologie des maladies Psychiatriques, UMR_S894 Institut National de la Santé et de la Recherche Médicale, Centre de Psychiatrie et NeurosciencesParis, France; ^2^Centre Hospitalier Sainte-Anne, Service Hospitalo Universitaire, Faculté de Médecine Paris Descartes, Université Paris DescartesParis, France

**Keywords:** cannabinoids, adolescence, long-term consequences, behavior, psychosis

## Abstract

Marijuana is the most widely used illicit drug among adolescents and young adults. Unique cognitive, emotional, and social changes occur during this critical period of development from childhood into adulthood. The adolescent brain is in a state of transition and differs from the adult brain with respect to both anatomy (e.g., neuronal connections and morphology) and neurochemistry (e.g., dopamine, GABA, and glutamate). These changes are thought to support the emergence of adult cerebral processes and behaviors. The endocannabinoid system plays an important role in development by acting on synaptic plasticity, neuronal cell proliferation, migration, and differentiation. Delta-9-tetrahydrocanabinol (THC), the principal psychoactive component in marijuana, acts as a partial agonist of the cannabinoid type 1 receptor (CB1R). Thus, over-activation of the endocannabinoid system by chronic exposure to CB1R agonists (e.g., THC, CP-55,940, and WIN55,212-2) during adolescence can dramatically alter brain maturation and cause long-lasting neurobiological changes that ultimately affect the function and behavior of the adult brain. Indeed, emerging evidence from both human and animal studies demonstrates that early-onset marijuana use has long-lasting consequences on cognition; moreover, in humans, this use is associated with a two-fold increase in the risk of developing a psychotic disorder. Here, we review the relationship between cannabinoid exposure during adolescence and the increased risk of neuropsychiatric disorders, focusing on both clinical and animal studies.

## Introduction

According to the Substance Abuse and Mental Health Services Administration, cannabis is the most commonly used drug of abuse among adolescents; in 2010, as many as 20% of all 16-year-olds surveyed reported using cannabis. Adolescence refers to the developmental time period between childhood and adulthood and is generally considered to occur from 12 to 17 years of age (Spear, 2000; Dahl, 2004). This period represents a critical phase in development, characterized by strong neurobehavioral plasticity and many maturation processes in the central nervous system, including myelination, synaptic pruning, volumetric growth, changes in receptor distribution, and programming of neurotrophic levels (Giedd et al., 1999; Spear, 2000; Bartzokis et al., 2001; Andersen, 2003). These changes occur primarily in the prefrontal cortex (PFC) and limbic regions (Chechik et al., 1999; Casey et al., 2000; Toga et al., 2006) and are believed to support the emergence of adult behavior and cognitive functions (Spear, 2000; Andersen, 2003).

Cannabinoid type 1 receptors (CB1R), type 2 receptors (CB2R), and their endogenous ligands (endocannabinoids; eCBs) anandamide (AEA) and 2-arachidonoyl glycerol (2-AG) are present and active during early brain development (Berghuis et al., 2005, 2007; Mulder et al., 2008). The eCB system modulates several neurodevelopmental processes, including the proliferation and differentiation of progenitor cells, neuronal migration, axonal guidance, fasciculation, positioning of cortical interneurons, neurite outgrowth, and morphogenesis (Harkany et al., 2007, 2008). In both adolescent humans and adolescent animals, the eCB system also undergoes functional development and changes (Rodriguez de Fonseca et al., 1993; Mato et al., 2003; Ellgren et al., 2008; Dalton and Zavitsanou, 2010; Eggan et al., 2010). In humans, CB1R expression increases dramatically from infancy to young adulthood in regions such as the frontal cortex, striatum and hippocampus (Mato et al., 2003). While these studies did not directly address specific phases of adolescence, studies in rodents have provided further information on changes in CB1R expression that may be regionally and temporally specific (Ellgren et al., 2008). CB1R expression increases progressively in the shell of the nucleus accumbens (NAcc) during adolescence, but decreases in the core of the NAcc during this same period (Ellgren et al., 2008). In the PFC, the expression of CB1R gradually decreases from mid-adolescence to late-adolescence with the greatest decreases observed in the cingulate, prelimbic, and infralimbic cortices (Ellgren et al., 2008; Heng et al., 2011). Concomitant to developmental changes in the CB1R, levels of AEA and 2-AG vary throughout adolescence in a region- and time-specific manner (Ellgren et al., 2008; Lee et al., 2013). There is an AEA spike in the NAcc during mid-adolescence; while in the PFC AEA progressively increases across adolescence (Ellgren et al., 2008). Conversely, levels of 2-AG are found to dramatically decline in the NAcc and PFC throughout adolescence (Ellgren et al., 2008). These distinct changes in CB1R and eCBs during adolescence suggest that this system plays both neurodevelopmental and morphogenic roles in the maturation of the central nervous system.

Given the importance of the eCB system in neurodevelopment, it is likely that externally induced changes in eCB signaling during adolescence can have profound long-term consequences on functioning in the adult brain. A large body of evidence obtained from both human studies and animal models suggests that exposure to cannabis during adolescence increases the likelihood of developing psychopathology in adulthood (Andreasson et al., 1987; Rubino et al., 2009b; Abush and Akirav, 2012; Renard et al., 2013).

The psychoactive effects of cannabis are due to the action of THC on CB1R receptors, which are present at high density in brain areas that play a role in processing emotional information, learning, and memory (e.g., the amygdala, PFC, and hippocampus) (Herkenham et al., 1990; Marsicano and Lutz, 1999). Perturbations in CB1R signaling in these brain regions have been linked to psychopathology and emotional dysregulation, which are common among neuropsychiatric disorders, including anxiety, depression, and schizophrenia (Degenhardt et al., 2003; Laviolette and Grace, 2006; Ahmad et al., 2013; Sanchez-Blazquez et al., 2014).

The aim of this review is to summarize published animal and human studies regarding the long-term consequences of cannabinoid exposure during adolescence, particularly the effects on cognitive functioning, emotional behavior, and the risk of developing a psychiatric disorder in adulthood. The relevant literature regarding the long-term consequences of cannabinoid exposure during adolescence in both human and animal studies is summarized in Table 1 and in Table 2, respectively.

**Table 1:** **Summary of the effect of adolescent cannabis use on behavior in human studies**.

**Behavior**	**First cannabis use (mean age)**	**Mean age at test**	**Cannabis use**	**Imaging method**	**Evaluation**	**Effect**	**Neurobiological correlates**	**References**
Cognition	16.8 ± 3.6	31.3 ± 7	Lifetime use	MRI/PET	–	–	Smaller whole brain	Wilson et al., 2000
			> 100 times				Smaller percent cortical gray matter	
							Larger percent white matter volumes	
	13.9 ± 1.1	21.8 ± 2.8	2.4 ± 1.6 joints a day	fMRI	Episodic memory	Encoding altered	↑ BOLD in left parahyppocampal gyrus	Becker et al., 2010a
	14.56 ± 0.53	21.44 ± 3.57	Lifetime use	fMRI	Behavioral inhibition	↓	↓ activity in anterior cingulate	Gruber et al., 2012
			> 2500 times					
	15.7 ± 0.9	17.8 ± 1	Lifetime use	MRI	Verbal fluency	↓	↓ cortical thickness in right caudal middle frontal, bilateral insula and bilateral superior frontal cortices	Lopez-Larson et al., 2011
			> 100 times				↑cortical thickness in the bilateral lingual, right superior temporal, right inferior parietal and left paracentral regions	
	13.1 ± 1.6	19.3 ± 0.8	5.8 joints a day	MRI	Verbal learning task	↓	Smaller volumes in hippocampus	Ashtari et al., 2011
	14.86 ± 0.31	17.7 ± 0.94	Lifetime use	MRI	Non- planning impulsivity	↑	↓ right medial orbital PFC	Churchwell et al., 2010
			> 500 times					
	n.s.	17.7 ± 0.7	Lifetime use	ASL	Verbal learning task	↓	↓ CBF in the left superior and middle temporal gyri, left insula, left and right medial frontal gyrus and left supramarginal gyrus	Jacobus et al., 2012
			> 200 days					
	14.1 ± 1.6	18.1 ± 0.7	Lifetime use	fMRI	Spatial Working memory	↓	↓ BOLD in DLPFC and occipital cortex	Schweinsburg et al., 2008
			> 100 times				↑BOLD posterior parietal cortex	
	16.3 ± 1.2	18.6 ± 0.8	>twice a month	–	Decision making impulsivity	↑	–	Solowij et al., 2012
Anxiety/Depressive disorders	14.5 ± 0.5	20.1 ± 0.5	Daily use	–	Anxiety	↑	–	Patton et al., 2002
	Follow-up study		Weekly use	–	Anxiety	↑	–	Degenhardt et al., 2013
	<14–18>	29						
	n.s.	18 ± 0.7	Lifetime use	MRI	Depressive symptoms	↑	↓ white matter volume	Medina et al., 2007b
			> 60 times					
	Follow-up study		Lifetime ever- use	–	Depressive symptoms	↑	–	Pedersen, 2008
	<13–17>	27						
Psychosis	Follow-up study		Lifetime ever- use	–	Schizophrenic symptoms	↑	–	Arseneault et al., 2002
	<15–18>	26						
	Birth cohort study		Lifetime ever- use	–	Schizophrenic symptoms	↑	–	Stefanis et al., 2004
	Before age of 16	18						

↓ , decrease;

↑ *, increase; MRI, magnetic resonance imaging; fMRI, functional magnetic resonance imaging; PET, positron emission tomography; ASL, arterial spin labeling; BOLD, blood oxygenation-level dependent; CBF, cerebral blood flow; PFC, prefrontal cortex; DLPFC, dorsolateral prefrontal cortex; n.s., not specified*.

**Table 2:** **Summary of the effect of adolescent cannabinoid exposure on behavior in various rat strains**.

**Behavior**	**Agonist CB1R**	**Adolescent treatment**	**Strain of rats**	**Sex**	**Test**	**Evaluation**	**Effect**	**Neurobiological correlates**	**References**
Sensorimotor gating	WIN55,212-2 (1.2 mg/kg)	PND40–65	Wistar	♂	Prepulse inhibiti on	Sensorimotor gating	↓		Schneider and Koch, 2003
								FOS protein expression altered in NAcc, caudate-putamen, hippocampus	Wegener and Koch, 2009
	WIN55,212-2 (2 mg/kg)	PND35–48	Lewis	♂	Prepulse inhibition	Sensorimotor gating	No effect	↑ number of surviving progenitor cells in striatum and PFC	Bortolato et al., 2014
								↑ striatal DOPAC	
Cognition	CP-55,940 (0.15/0.20/0.30 mg/kg)	PND29–50	Wistar Lister Hooded	♂	Object location	Spatial working memory	↓		Renard et al., 2013
	CP-55,940 (0.15/0.20/0.30 mg/kg)	PND29–50	Wistar Lister Hooded	♂	Object Recognition	Short-term memory	↓		Renard et al., 2013
	Δ9-THC (2.5/5/10 mg/kg)	PND35–45	Sprague Dawley	♀	Object Recognition	Short-term memory	↓	↓ GAD67 in PFC	Zamberletti et al., 2014
	CP-55,940 (0.15/0.20/0.30 mg/kg)	PND30–51	Wistar	♀♂	Object Recognition	Short-term memory	↓		O'Shea et al., 2006
	WIN55,212-2 (1.2 mg/kg)	PND40–65	Wistar	♂	Object Recognition	Short-term memory	↓		Schneider and Koch, 2003
	Δ9-THC (5 mg/kg)	P ND30–51	Sprague Dawley	♀♂	Water maze	Working and spatial memories	No effect		Cha et al., 2007
	Δ9-THC (2.5/5/10 mg/kg)	PND35–45	Sprague Dawley	♂	Radial maze	Spatial working memory	↓	Alteration of neuronal morphology and synaptic proteins in hippocampus	Rubino et al., 2009b
				♀				Modifications in level expression of:	Rubino et al., 2009a
								-neuronal plasticity proteins in PFC,	
								-cytosqueletal and structural proteins in PFC	
	Δ9-THC (5 mg/kg)	PND32–48	Wistar	♀	Object Recognition	Short-term memory	↓	Modifications in expression levels of:	Quinn et al., 2008
								-cytosqueletal and structural proteins in hippocampus,	
								-proteins related to degenerative and oxidative changes in hippocampus	
	CP-55,940 (0.4 mg/kg)	PND28–38	Wistar	♀♂	Water maze	Working and spatial memories	No effect	↑ PSA-NCAM in hippocampus	Higuera-Matas et al., 2009
Emotions	CP-55,940 (0.4 mg/kg)	PND35–45	Wistar	♂	Open Field	Anxiety	No effect		Biscaia et al., 2003
				♀	Elevated Plus Maze	Anxiety	↓		
	Δ9-THC (5 mg/kg)	PND32–48	Wistar	♂	Social interaction	Social behaviors	↓	Modifications in expression levels of:	Quinn et al., 2008
								-cytosqueletal and structural proteins in hippocampus,	
								-proteins related to degenerative and oxidative changes in hippocampus	
	Δ9-THC (2.5/5/10 mg/kg)	PND35–45	Sprague Dawley	♀	Social interaction	Social behaviors	↓	↓ GAD67 in PFC	Zamberletti et al., 2014
	Δ9-THC (2.5/5/10 mg/kg)	PND35–45	Sprague Dawley	♀	Forced Swimming test	Depressive-like disorders	↑	↓ GAD67 in PFC	Zamberletti et al., 2014
	Δ9-THC (2.5/5/10 mg/kg)	PND35–45	Sprague Dawley	♀	Forced Swimming test	Depressive-like disorders	↑	↓ CB1R function in NAcc, Amygdala and VTA,	Rubino et al., 2008
								↓ P-CREB in Hippocampus and PFC,	
								↑P-CREB in NAcc	
	CP-55,940 (0.4 mg/kg)	PND28–38	Wistar	♀♂	Elevated Plus Maze	Anxiety-like disorders	↑	↑ PSA-NCAM in hippocampus	Higuera-Matas et al., 2009

↓ , decrease;

↑ *, increase; DOPAC, 3,4-dihydroxyphenylacetic acid; GAD67, glutamic acid decarboxylase 67; PSA-NCAM, polysialylated form of the neural cell adhesion molecule; PFC, prefrontal cortex; CB1R, cannabinoid receptors type 1; VTA, ventral tegmental area; NAcc, nucleus accumbens*.

*Δ9-THC acts as a partial agonist for CB1R (K_i_ = 5.05–80.3 nM). CP-55,940 and WIN55,212-2 are full CB1R agonists. CP-55,940 has a greater affinity and selectivity for the CB1R (K_i_ = 0.5–5 nM) than THC and WIN55,212-2 (K_i_ = 1.89–123 nM) (Pertwee, 1999)*.

## Long-term effects of cannabis use during adolescence in humans

### Cognitive disorders and neuroimaging alterations

Chronically using cannabis before the age of 17 causes more severe cognitive consequences compared to chronic use later in adolescence. Indeed, converging lines of evidence suggest that chronic use before the age of 17 is associated with deficits in working memory (Schweinsburg et al., 2008, 2010; Becker et al., 2010b), attention (Ehrenreich et al., 1999; Meier et al., 2012; Dougherty et al., 2013), decision-making (Dougherty et al., 2013), visual search (Huestegge et al., 2002), overall and verbal IQ (Pope et al., 1997; Meier et al., 2012), executive functioning (Medina et al., 2009; Becker et al., 2010a; Fontes et al., 2011; Solowij et al., 2012), visuospatial memory (Pope et al., 1997), cognitive inhibition (Fontes et al., 2011), and impulsivity (Dougherty et al., 2013). The magnitude of these deficits is proportional to the frequency, dose, and age at onset of use (Medina et al., 2007a; Schweinsburg et al., 2008, 2010; Becker et al., 2010a; Fontes et al., 2011; Meier et al., 2012).

Although the precise mechanism through which cannabis impairs cognition remains unknown, structural abnormalities have been measured in long-term, heavy cannabis users.

Magnetic resonance imaging (MRI) and positron emission tomography (PET) studies have reported reduced overall cortical gray matter and increased white matter volume in adolescent cannabis users compared to users who began using cannabis in adulthood (Amen and Waugh, 1998; Wilson et al., 2000). Cannabis use during adolescence has also been attributed to increased white matter diffusivity in the PFC compared to later use (Becker et al., 2010a). Functional MRI (fMRI) studies have revealed abnormal activation in the PFC and parietal brain regions of adolescent cannabis users (Jager et al., 2010; Becker et al., 2010a; Gruber et al., 2012). Other groups have also reported decreased cortical thickness in the right superior PFC, bilateral insula, and bilateral superior frontal cortices; increased cortical thickness in the lingual, temporal, inferior parietal, and paracentral regions (Lopez-Larson et al., 2011); and decreased volume in the right medial orbitofrontal cortex (Churchwell et al., 2010) and bilateral hippocampus (Yucel et al., 2008; Ashtari et al., 2011) of adolescent cannabis users without a comorbid psychiatric condition compared to adolescents who do not use cannabis. These structural changes have been associated with increased executive dysfunction (Medina et al., 2009; Churchwell et al., 2010) and verbal memory deficits (Ashtari et al., 2011). In addition, adolescent users of cannabis have reduced cerebral blood flow in the temporal, insular, and PFC regions, and these reductions in blood flow are associated with cognitive deficits (Jacobus et al., 2012). Moreover, converging evidence suggests the presence of abnormal activation patterns in the PFC, limbic region, parietal region, and cerebellum (Schweinsburg et al., 2008; Becker et al., 2010a; Lopez-Larson et al., 2012; Vaidya et al., 2012) in adolescent users of cannabis compared to subjects who do not use cannabis. Interestingly, using diffusion-weighted MRI and connectivity mapping, Zalesky et al. (2012) showed that axonal connectivity was impaired in the right fimbria and the corpus callosum, two structures that contain abundant levels of cannabinoid receptors in the developing brain (Romero et al., 1997; Molina-Holgado et al., 2002). Zalesky and colleagues also demonstrated that the age at which regular cannabis use begins is a key factor in determining the severity of the resulting microstructural changes in white matter (Zalesky et al., 2012).

Taken together, the above studies suggest that chronic cannabis use during adolescence can cause long-term structural changes that are associated with decreased neuronal efficiency in brain regions that play a central role in learning and memory.

### Anxiety disorders

Anxiety disorders are the most common complications that arise from chronic heavy cannabis use. Whereas the lifetime prevalence for anxiety disorder on general population is estimated around 6–17% (Kedzior and Laeber, 2014), this prevalence is increased in cannabis users with a prevalence up to 20% (Reilly et al., 1998). A broad and recent meta-analysis (Kedzior and Laeber, 2014) shows that anxiety is significantly positively associated with the consumption (Odds ratio = 1.24) and misuse of cannabis (Odds ratio = 1.68). However, only a few studies have examined the relationship between adolescent cannabis use and long-term anxiety disorders. Cannabis uses during adolescence can double the risk of developing anxiety-related symptoms in adulthood, particularly if the onset of use was initiated before the age of 15. Moreover, girls are more likely than boys to develop these symptoms (Patton et al., 2002; Hayatbakhsh et al., 2007).

A recent study examined the relationship between cannabis use and mental health between the ages of 15 and 29. The authors found that heavy cannabis use during adolescence was associated with an increased risk of developing an anxiety disorder later in life, even if the individual no longer uses cannabis in adulthood (Degenhardt et al., 2013).

### Depressive disorders

In many countries, a growing body of evidence supports an association between cannabis use and depression among young people. Among cannabis users, the prevalence of depressive disorders is 25%; approximately half of these depressive disorders are major depression, and the other half are severe mood disorders (Chabrol et al., 2008). This risk can increase by five-fold, depending on gender (women seem to be more susceptible than men) and the age at which cannabis use begins (Grant and Pickering, 1998; Green and Ritter, 2000).

A study conducted among Australians between 13 and 17 years of age found that adolescents who use cannabis are three times more likely to meet the criteria for depression later in life compared to adolescents who never used cannabis (Rey et al., 2002). Another study found that 30% of adolescents who chronically use cannabis between the ages of 15 and 17 develop depressive symptoms by the age of 21 (Fergusson et al., 2003). Van Laar and colleagues confirmed this observation and also reported that frequent cannabis use increases the risk of developing a depressive disorder (van Laar et al., 2007). In addition, Hayatbakhsh and colleagues demonstrated that adolescents under the age of 15 who frequently use cannabis are more likely to report symptoms of anxiety and depression in early adulthood, particularly when the researchers took into account cumulative exposure to cannabis and potentially significant confounding factors such as maternal smoking and alcohol consumption (Hayatbakhsh et al., 2007). Finally, a longitudinal study that was conducted in young Norwegians and followed over a 13-year period (from their early teens to their late teens) showed a dose-dependent relationship between chronic cannabis consumption and suicidal tendencies (i.e., thoughts and attempts) later in life (Pedersen, 2008). Taken together, these longitudinal studies suggest that early onset and regular use of cannabis increase the risk of depression later in life. However, some researchers suggest that both environmental factors and genetic predisposition play a role in this causal association (Lynskey et al., 2004; Vinod and Hungund, 2006). Indeed, Lynskey and colleagues observed that twins who were discordant for cannabis dependence were more likely to develop suicidal ideation or attempted suicide than their non-cannabis-dependent co-twins. They also reported that twins discordant for early marijuana initiation (before the age of 17) were more likely to attempt suicide than their non-early-cannabis use co-twins (Lynskey et al., 2004). Furthermore, post-mortem studies revealed that the density of CB1R was higher in the PFC of patients with depression who died by suicide than controls (Vinod and Hungund, 2006).

Finally, increased depressive symptoms in adolescent users of cannabis were shown to be associated with smaller global white matter volume (Medina et al., 2007b), suggesting that cannabis use during adolescence may disrupt white matter connections between brain regions that play a role in mood regulation.

These studies put forward that pre-existing genetic factors may predispose an individual to depression, and that these factors may be revealed by early cannabis use. Future studies are clearly needed in order to elucidate the neurobiological mechanisms that underlie the long-lasting effects of cannabis use on the eventual onset of depressive disorders.

### Psychosis: focus on schizophrenia

The psychotic symptoms that have been associated with the use of cannabis (i.e., the so-called cannabis-induced psychosis) include a “loss of control,” thought disturbances, feelings of unreality, apprehension, fear and paranoia, depersonalization, dysphoria, difficulty concentrating, hallucinations, and other perceptual alterations (Hall and Degenhardt, 2000; Degenhardt and Hall, 2002). The first longitudinal study to demonstrate an association between adolescent cannabis use and schizophrenia in later life was conducted in young healthy Swedish subjects (Andreasson et al., 1987). The authors found that heavy cannabis use at age 18 led to a six-fold increase in the risk of developing schizophrenia 15 years later. In addition, an 11-year longitudinal study of subjects who did not present with pre-existing psychosis found that cannabis use was associated with an increased risk of developing schizophrenic symptoms (Arseneault et al., 2002). This study also revealed that early cannabis consumption (i.e., at age 15) increased the risk of developing schizophrenic symptoms at age 26 by a factor of four compared to cannabis consumption after age 18. According to the authors, 8–13% of the patients in their study might never have developed schizophrenic symptoms had they not used cannabis (Arseneault et al., 2004). This strong association between adolescent cannabis use and psychotic disorders later in life was confirmed by Stefanis and colleagues, who found that both positive and negative psychotic symptoms were more strongly associated with an onset of cannabis use before the age of 16, regardless of the frequency or duration of use (Stefanis et al., 2004).

There is still a debate on whether adolescent cannabis use triggers the onset of schizophrenia in genetically vulnerable individuals or whether individuals with pre-existing vulnerability for psychosis are more likely to use cannabis as a means of self-medication to alleviate some early psychotic symptoms (Dixon et al., 1990; Mueser et al., 1992; Ferdinand et al., 2005; Degenhardt et al., 2007). This proposed self-medication explanation does not account for the association between cannabis use during adolescence and the development of psychosis later in life, as this relationship is observed more often in the absence of psychological distress, and psychological distress does not necessarily predict cannabis use (Stefanis et al., 2004; Henquet et al., 2005).

In addition, there is overwhelming consensus within the literature citing a lack of evidence for the self-medication hypothesis, given that no relationship between early psychotic symptoms and an increased risk of later cannabis use has been reported (Patton et al., 2002; Tournier et al., 2003; Verdoux et al., 2003, 2005).

Yet, only a small minority of cannabis users develop psychotic symptoms. It is therefore likely that both environmental factors and genetic predisposition play a role in this causal association. Consistent with this notion, among patients with schizophrenia, hypersensitivity to the psychotomimetic effects of cannabis is associated with early cannabis exposure and a family history of psychosis (Arendt et al., 2008; Goldberger et al., 2010); moreover, a recent study of a large sample of students found that sensitivity to the psychotomimetic effects of cannabis appears to be an intrinsic feature present since the first exposure to cannabis (Krebs et al., 2014).

## Neurobiological basis of the link between cannabis use and schizophrenia

### Cannabinoid receptors

Studies in humans support CB1R dysfunction in certain brain regions of schizophrenic patients, specifically in the cortical regions that play a role in cognition and memory (i.e., the anterior and posterior cingulate cortices and the dorsolateral PFC), two functions that are severely compromised in schizophrenia. Indeed, post-mortem analyses revealed that the density of CB1R receptors was higher in the dorsolateral PFC and cingulate cortices (both anterior and posterior) of schizophrenic patients compared to controls, and these changes were apparently independent of recent pre-mortem cannabis use (Dean et al., 2001; Zavitsanou et al., 2004; Newell et al., 2006).

### Endocannabinoids

Clinical studies suggest that in addition to altering CB1R function, schizophrenic patients have changes in their eCB levels, particularly AEA. Indeed, AEA levels are higher in the cerebrospinal fluid of non-medicated paranoid schizophrenic patients than in healthy subjects, irrespective of recent cannabis use (Leweke et al., 1999). Interestingly, other lipid molecules, including oleoylethanolamide and palmitoylethanolamide, were not increased in these patients, excluding the likelihood of schizophrenia-related changes in general lipid signaling (Giuffrida et al., 2004).

Treating schizophrenic patients with classic antipsychotics (i.e., dopamine D2 receptor antagonists) lowers AEA levels to normal. Because paranoid schizophrenia is characterized primarily by predominantly positive symptoms due to hyper-dopaminergic neurotransmission (Oades et al., 2002), AEA has been suggested to play an adaptive role, counteracting the dopaminergic abnormalities in schizophrenia, thus reinforcing the existence of dysregulated AEA signaling in schizophrenia. Another study by the same group found that frequent cannabis use down-regulates AEA levels in the cerebrospinal fluid of schizophrenic patients, but not healthy controls (Leweke et al., 2007). These results indicate that frequent cannabis exposure may lead to the down-regulation of AEA signaling (by decreasing AEA biosynthesis and/or increasing AEA degradation) in the central nervous system of schizophrenic patients, but not healthy patients. This down-regulation of AEA might disrupt AEA's control over dopaminergic neurotransmission, thereby precipitating psychosis. Accordingly, alterations in AEA signaling might be an important component of the putative mechanism through which cannabis precipitates psychotic symptoms.

### Genetic vulnerability to cannabis

A growing body of data in recent years supports the notion that genetics plays a clear role in the association between early cannabis exposure and an increased risk of developing schizophrenia. Following the first published report of an association between a CNR1 receptor polymorphism and cannabis abuse in schizophrenia (Leroy et al., 2001), a second study of a Japanese cohort found that a polymorphism in the *CNR1* gene (which encodes the CB1R receptor) may be associated with an increased risk of developing hebephrenic (i.e., disorganized) schizophrenia (Ujike et al., 2002). This form of schizophrenia is characterized primarily by negative symptoms that resemble the amotivational features commonly observed following chronic cannabis consumption. However, the current state of the literature does not suggest that this polymorphism would be a vulnerability factor for hebephrenic schizophrenia and further studies are needed to confirm *CNR1* polymorphisms as a genetic risk for hebephrenic schizophrenia.

A more recent study evaluated interactions between *CNR1* polymorphisms, heavy cannabis use, cerebral volume and cognitive function (Ho et al., 2011). Authors compared schizophrenia patients with heavy cannabis use and schizophrenia patients without heavy cannabis use. First, they observed that schizophrenia patients with cannabis abuse had smaller fronto-temporal white matter volumes than patients without heavy cannabis use. In addition, they found that schizophrenia patients with specific *CNR1* polymorphisms (specifically rs12720071 SNP G-allele carriers) were more vulnerable to the impact of heavy cannabis use, as they showed greater white matter volume decrease and cognitive impairment than patients without heavy cannabis use. These results are suggestive of gene-environment interactions for conferring phenotypic abnormalities in schizophrenia (Ho et al., 2011).

The relationship between adolescent cannabis use and psychotic symptoms may also be attributed to a functional polymorphism in the catechol-*O*-methyltransferase (*COMT*) gene, which encodes an enzyme that degrades catecholamines such as dopamine (DA). The human *COMT* gene has two allelic variants that code for either a valine or a methionine at codon 158. The functional COMT valine 158 variant catabolizes synaptic DA faster than the COMT methionine 158 variant (Lachman et al., 1996). Caspi and colleagues found that carriers of the COMT valine 158 allele who use cannabis are more likely to exhibit psychotic symptoms and develop a schizophrenic disorder; in contrast, cannabis consumption had no such effect on individuals with two copies of the methionine variant (Caspi et al., 2005). Finally, increased COMT activity may underlie the decrease in synaptic DA levels that contributes to the development of cognitive disorders (Chen et al., 2004).

Polymorphisms in the *AKT1* gene may play a role in psychosis induced by early cannabis use. AKT1 is a serine/threonine kinase that helps regulate dopaminergic signaling cascades. Cannabinoids can activate the AKT1 pathway via CB1R receptors. Indeed, Decoster and colleagues recently reported that a genetic variation in the *AKT1* gene may mediate cannabis-associated effects on the expression of psychosis via a cannabinoid-regulated AKT1/GSK-3 signaling mechanism that lies downstream of the dopamine D2 receptor (Decoster et al., 2011).

Finally, a recent study of nearly 1200 young healthy students revealed that the psychotomimetic effects at first cannabis use were associated with *CNR1* variants but not with *COMT* or *AKT1* variants (Krebs et al., 2014). This finding supports the notion that individual variability in the psychotomimetic effect of cannabis may be attributed to specific genetic backgrounds that influence an individual's first response to cannabis, potentially revealing increased risk of developing psychosis later in life in those individuals (Krebs et al., 2014).

Taken together, these studies confirm that cannabis use—particularly during adolescence—can contribute to the emergence of psychotic disorders in genetically vulnerable individuals, supporting the “two-hit” hypothesis, which posits that both genetics and environmental factors encountered early in life increase the individual's risk of developing a psychiatric disorder (see below).

## Studying the long-term effects of cannabinoid exposure during adolescence using animal models

**Figure 1:**
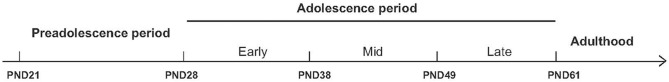
**Schematic overview of the various stages of adolescence in rats**. To model the effects of cannabinoid exposure at different developmental stages, rats can be treated chronically throughout the entire adolescent period (from PND28 through PND61) or during specific stages of adolescence, including early adolescence (beginning at PND28), mid-adolescence (beginning at PND38), or late adolescence (beginning at PND49). The long-term effects of adolescent cannabinoid exposure can then be measured in adulthood. PND, postnatal day.

Animal models are useful tools for investigating the long-term behavioral effects of cannabis exposure during adolescence. To develop a suitable animal model, one must first take into consideration the obvious differences in developmental ontogeny between the animal species and human patients. Adolescence has a broad developmental border in both humans and animals. In rodents, adolescence ranges from postnatal day 28 (PND28) to PND42 (Spear, 2000), and it can be subdivided into specific phases such as early adolescence (beginning at PND28), mid-adolescence (beginning at PND38), and late adolescence (beginning at PND49) (Figure 1).

The relevant literature regarding the long-term consequences of cannabinoid exposure during adolescence in rats is summarized in Table 2.

### Cognitive impairments

Chronically exposing adolescent rats (of either gender) to CB1R agonists (including THC, WIN55,212-2, and CP55,950) induces short-term memory impairment measured using the object recognition task (Schneider and Koch, 2003; O'Shea et al., 2006; Quinn et al., 2008; Schneider et al., 2008; Llorente-Berzal et al., 2013; Renard et al., 2013; Zamberletti et al., 2014). In contrast, chronic cannabinoid exposure to adult rats has no long-term significant effect on this cognitive function (Schneider and Koch, 2003; Quinn et al., 2008; Renard et al., 2013). However, one study demonstrated that chronic THC treatment in adult mice impairs performance in the object recognition task for 4 days following the last THC injection and these deleterious effects were shown to be prevented by a pre-treatment with Temsirolimus, an mTOR inhibitor, suggesting a role for mTOR in the THC-induced long-lasting memory impairment (Puighermanal et al., 2013).

Similarly, deficits in spatial working memory have been observed in adult rats that were exposed to CB1R agonists during adolescence; these deficits were detected using the radial maze task (Rubino et al., 2009a,b) and the object location task (Abush and Akirav, 2012; Renard et al., 2013). With respect to the potential neurobiological substrates that may underlie this phenomenon, these cognitive deficits have been correlated to reduced expression of several proteins in the hippocampus and in the PFC, including synaptic plasticity proteins (PSD95 and synaptophysin), cytoskeletal and structural proteins related to degenerative and oxidative changes, NMDA receptors, glutamic acid decarboxylase 67 (GAD 67), glial fibrillary acidic proteins (GFAP), and activity-regulated cytoskeletal-associated protein (Arc) (Quinn et al., 2008; Rubino et al., 2009a,b; Llorente-Berzal et al., 2013; Zamberletti et al., 2014). In addition, adult rats that were treated with cannabinoids during adolescence have reduced total dendritic length, arborization, and spine numbers in the dentate gyrus (Rubino et al., 2009b). Interestingly, exposure to cannabinoids during adolescence does not appear to induce long-lasting memory impairments in adult rats (measured using the Morris water maze) of either gender (Cha et al., 2007; Higuera-Matas et al., 2009). Overall, the deleterious cognitive effects of early cannabinoid exposure point to long-lasting impairments in short-term—rather than long-term—information processing. However, further research is needed in order to understand these differential changes in the temporal aspects of memory.

Taken together, these findings obtained from many animal studies support the results obtained from human studies and confirm that adolescence is a highly critical period with long-term downstream effects on cognitive processing.

### Anxiety-like behavior

With respect to anxiety, the results obtained from animal studies are less consistent than cognitive function, although this lack of consistency may be due to the specific developmental period in which cannabinoids are administered and/or the behavioral test used to measure anxiety. Chronic treatment with the cannabinoid agonist CP-55,940 from PND35 to PND45 decreased anxiety levels assessed using the open-field test and the elevated plus maze (Biscaia et al., 2003). However, no changes in plasma corticosterone levels were found in the adult rats, suggesting “normal” function and activity of the hypothalamo-hypophyso-cortico-adrenal axis. In addition, chronic treatment with various CB1R agonists (e.g., THC, CP-55,940, and WIN55,212-2) decreased anxiety levels in adult rats assessed using the social interaction test (O'Shea et al., 2006; Quinn et al., 2008; Zamberletti et al., 2014).

Conversely, treating rats chronically with CP-55,940 from PND28 to PND38 or with THC from PND28 to PND45 led to an increase in anxiety levels at adulthood, measured using the elevated plus maze (Higuera-Matas et al., 2009) or the open-field test, respectively (Llorente-Berzal et al., 2013). Similarly, chronically treating rats with high doses of WIN55,212-2 from PND30 to PND50 induced anxiety-like effects at adulthood, measured using the novelty-suppressed feeding test (Bambico et al., 2010).

### Depressive-like behavior

Experimental studies revealed that exposing adolescent rats to cannabinoids leads to an immediate dysregulation of emotional processes and induces a depressive-like phenotype later in life. Treating adolescent (PND35–PND45) rats with WIN55,212-2 or THC led to decreased sucrose preference (a measure of anhedonia) in adulthood (Rubino et al., 2008; Bambico et al., 2010). Subjecting adolescent cannabinoid-treats rats to the forced-swim test also revealed a depressive phenotype, and these effects appear to be stronger in female rats (Rubino et al., 2008; Bambico et al., 2010; Zamberletti et al., 2014). These findings suggest that cannabinoid exposure during adolescence may affect the susceptibility to develop a mood disorder later in life, and these animal models appear to recapitulate the gender differences that are well-documented among human patients with depression. Investigating the neurobiological mechanisms that underlie the change in emotion induced by adolescent cannabinoid exposure, Rubino and colleagues found a decrease in CB1R expression in the amygdala, ventral tegmental area, and NAcc, thereby demonstrating a role for CB1R receptors in the regulation of emotional response (Rubino et al., 2008). These changes in CB1R expression were accompanied by changes in the levels of CREB protein in the hippocampus, NAcc, and PFC (Rubino et al., 2008). More recently, Bambico and colleagues reported that treating adolescent rats—but not adult rats—with WIN55,212-2 alters midbrain neuronal firing (Bambico et al., 2010). Specifically, adolescent cannabinoid treatment resulted in hyperactivity of noradrenergic neurons concomitant with hypoactivity of serotonergic neurons (Bambico et al., 2010). Such neuroadaptations are consistent with enhanced depressive-like behavior due to cannabinoid exposure during adolescence.

### Psychotic-like behavior

To date, animal studies regarding psychotic symptoms have focused on disruptions in ^**^prepulse inhibition (PPI). Impaired PPI is widely accepted as an endophenotype of schizophrenia with high translational validity (Braff et al., 2001; Perry et al., 2001).

Chronically treating adolescent rats (PND40–PND65) with the cannabinoid agonist WIN55,212-2 induced long-lasting sensorimotor gating impairment in adulthood (i.e., 85 days after treatment), measured using the PPI test (Schneider and Koch, 2003; Wegener and Koch, 2009); in contrast, chronically treating adult rats did not affect PPI. These deficits were correlated with changes in basal neuronal activity (i.e., c-Fos activity) in the NAcc, amygdala, caudate putamen, and hippocampus (Wegener and Koch, 2009). In contrast, other groups reported no changes in PPI after chronically exposing adolescent rats (PND35–PND45) to THC (Bortolato et al., 2014). This discrepancy may be due to the adolescent period in which the rats were treated and/or the cannabinoid compound that was administered. These differences in results may also reflect differences in the genetic backgrounds of the rats used in these studies (for example, Bortolato and colleagues used Lewis rats, whereas Schneider and colleagues used Wistar rats). Indeed, strain differences have been found to play a critical role in modulating the acoustic startle reflex and sensorimotor gating in rats (Hsieh et al., 2006).

### The “two-hit” neurodevelopmental animal model of schizophrenia

As discussed above, the “two-hit hypothesis” of schizophrenia posits that a prenatal genetic or environmental “first hit” increases the individual's vulnerability to a “second hit” later in life, thereby significantly increasing the risk of developing schizophrenic symptoms (Bayer et al., 1999). To test this hypothesis, *COMT*-knockout mice (the “first hit”) were exposed to THC during adolescence (the “second hit”); this two-hit approach induced long-lasting impairments in exploration, spatial working memory, and anxiety (O'Tuathaigh et al., 2010), as well as deficits in PPI, habituation to the acoustic startle response, sociability, and social novelty preference (O'Tuathaigh et al., 2012). Exposing adolescent *COMT*-knockout mice to THC also decreased the size of dopaminergic and GABAergic cells in the ventral tegmental area and PFC, respectively, and increased CB1R expression in the hippocampus (Behan et al., 2012). These results reinforce the notion that an individual's risk of developing schizophrenia may be influenced by interactions between genetic-based changes in DA catabolism and cannabis exposure. These finding also suggest how *COMT* deletion and adolescent cannabis use may work together to modulate the function of neurotransmitter systems implicated in schizophrenia.

Other studies have also revealed an interesting interaction between cannabis exposure and a mutation in the *neuregulin 1* (*Nrg1*) gene. Mice that are heterozygous for the transmembrane domain Nrg1 mutant (*Nrg1* HET mice) appeared to be more sensitive to the behavioral effects of acute THC exposure than their wild-type littermates (Boucher et al., 2007a; Long et al., 2010). Following chronic exposure to THC, *Nrg* HET mice developed tolerance to cannabinoid-induced hypothermia and locomotor suppression more rapidly than wild-type mice (Boucher et al., 2011). Conversely, wild-type mice (but not *Nrg1* HET) mice developed tolerance to cannabinoid-induced anxiety, whereas *Nrg1* HET mice showed a persistent anxiogenic response to chronic cannabinoid exposure. Cannabinoid exposure (either acute or chronic) also selectively induced the expression of Fos transcription factors in the lateral septum of *Nrg1* HET mice, but not wild-type mice (Boucher et al., 2007b, 2011). In addition, chronically exposing adolescent *Nrg1* HET mice to THC exacerbated their hyperlocomotor phenotype and induced differential effects in the density of NMDA, CB_1_, and 5-HT_2A_ receptors in brain regions that are relevant to schizophrenia, particularly the hippocampus (Long et al., 2013). More recently, these researchers expanded their previous findings, reporting that chronic adolescent exposure to THC induces the differential expression of proteins involved in NMDA receptor trafficking to the synaptic membrane, lipid raft stabilization of synaptic NMDA receptors, and homeostatic responses that dampen excitotoxicity (Spencer et al., 2013). These observations suggest an interaction between the cannabinoid system and Nrg1 signaling and indicate that altered expression of the *Nrg1* gene can modulate the behavioral consequences of THC exposure during adolescence.

Similarly, Schneider and Koch (2007) reported that adolescent cannabinoid exposure induces stronger deficits in both short-term memory and social behavior in rats that received a neonatal PFC lesion. In addition, exposure to THC during adolescence exacerbated (i) the short-term memory deficit in rats that were chronically pretreated with PCP (measured using the object recognition test) (Vigano et al., 2009), and (ii) the disruption in PPI in socially isolated rats (Malone and Taylor, 2006).

Taken together, these studies of the interaction between genetic factors and environmental factors help elucidate the putative mechanisms underlying the etiopathogenesis of schizophrenia and may facilitate the discovery of new therapeutic targets.

## Conclusions

A considerable body of evidence obtained from both human studies and animal models suggests that cannabis use during adolescence increases the risk of developing a psychiatric disorder in adulthood, including anxiety, depression, and schizophrenia. The psychiatric disorders discussed in this review are multifactorial in origin, and the transition from adolescent cannabis use to subsequent psychiatric illness may also involve both genetic factors and environmental factors. The developmental period of initiation, the frequency, and the duration of cannabis use, as well as any underlying psychiatric pathology, may all play a critical role in the development of a psychiatric disorder. In addition, compelling evidence suggests that some adolescents are more susceptible to the long-term effects of cannabis use than others, and this may be due to differences in genetic vulnerability, including polymorphisms in the genes that play a role in the development of psychiatric diseases. However, whether early cannabis use is related to a pre-existing pathology that is exacerbated by drug use remains an open question.

Disrupting the eCB system during development may affect several neurotransmitter systems. Indeed, CB1R receptors are presynaptic and are expressed primarily in GABAergic interneurons and pyramidal neurons (Marsicano and Lutz, 1999; Manzoni and Bockaert, 2001; Morozov and Freund, 2003). By modulating the release of GABA and glutamate, CB1R receptors help prevent excess neuronal excitation and inhibition (Marsicano et al., 2003; Kano et al., 2009; Katona, 2009). Repeated excessive CB1R stimulation by THC during adolescence may shift the balance of GABAergic inhibitory input on pyramidal neurons. We can therefore speculate that exposure to cannabinoids during adolescence might interfere with the development of neuronal processes in the still-developing adolescent brain, thus leading to changes that ultimately affect brain connectivity, function, and behavior.

Longitudinal studies using neuroimaging and genetic approaches to evaluate adolescents prior to the start of chronic cannabis use are needed. In addition, studies using animal models are needed in order to further investigate the role of the endocannabinoid system in adolescence, as well as the molecular and neurochemical mechanisms that underlie the deleterious effects of cannabinoid exposure during adolescence. Such studies would greatly enhance our understanding of the propensity for adolescent cannabis use to facilitate the development of psychiatric disease later in life.

### Conflict of interest statement

The authors declare that the research was conducted in the absence of any commercial or financial relationships that could be construed as a potential conflict of interest.
